# Total Globulin Fraction at Diagnosis Could Forecast All-Cause Mortality during the Disease Course in Patients with Antineutrophil Cytoplasmic Antibody-Associated Vasculitis

**DOI:** 10.3390/jcm12124170

**Published:** 2023-06-20

**Authors:** Jang-Woo Ha, Sung-Soo Ahn, Jason-Jungsik Song, Yong-Beom Park, Sang-Won Lee

**Affiliations:** 1 Division of Rheumatology, Department of Internal Medicine, Yonsei University College of Medicine, Seoul 03722, Republic of Korea; hjwnmk@yuhs.ac (J.-W.H.); saneth@yuhs.ac (S.-S.A.); jsksong@yuhs.ac (J.-J.S.); yongbpark@yuhs.ac (Y.-B.P.); 2 Institute for Immunology and Immunological Diseases, Yonsei University College of Medicine, Seoul 03722, Republic of Korea

**Keywords:** globulin, fraction, mortality, antineutrophil cytoplasmic antibody, vasculitis

## Abstract

Total globulin fraction (TGF) is calculated by subtracting serum albumin levels from serum total protein levels. The present study examined whether TGF at diagnosis could forecast all-cause mortality during the disease course in patients with antineutrophil cytoplasmic antibody (ANCA)-associated vasculitis (AAV). The present study included 283 patients with AAV. The variables at AAV diagnosis such as demographic data, AAV-specific data including the Birmingham vasculitis activity score (BVAS), five-factor score (FFS), and laboratory data including ANCA, erythrocyte sedimentation rate (ESR), and C-reactive protein (CRP) were collected. The number of deceased patients during the follow-up duration based on all-cause mortality was counted. The median age of the 283 AAV patients was 60 years, and 35.7% were men. ANCAs were detected in 228 patients, and the median TGF was 2.9. A total of 39 patients (13.8%) died within a median follow-up duration of 46.9 months. TGF at AAV diagnosis was significantly correlated with ESR and CRP rather than AAV activity. Patients with ANCA positivity exhibited a significantly higher median TGF at AAV diagnosis than those without. Patients with TGF ≥ 3.1 g/dL at AAV diagnosis exhibited a significantly lower cumulative survival rate than those without. Furthermore, in the multivariable Cox hazards model analysis, TGF ≥ 3.1 g/dL (hazard ratio 2.611) was independently associated with all-cause mortality, along with age, male sex, and body mass index. The present study is the first to demonstrate that TGF at AAV diagnosis can forecast all-cause mortality during the disease course in AAV patients.

## 1. Introduction

Antineutrophil cytoplasmic antibody (ANCA)-associated vasculitis (AAV) is a systemic vasculitis that affects small-sized vessels primarily and medium-sized arteries infrequently [1,2]. AAV is characterised by necrotising vasculitis on histology and is classified into three subtypes based on the presence of granulomatous formation and eosinophilic infiltration: microscopic polyangiitis (MPA), granulomatosis with polyangiitis (GPA), and eosinophilic granulomatosis with polyangiitis (EGPA) [1]. The rates of all-cause mortality in AAV patients are somewhat higher than those in patients with other systemic vasculitides; a previous study reported that the rate of all-cause mortality was 38.4 per 1000 patients per year, and another previous study reported an age-standardised mortality rate of 0.53 deaths per 1 million people [3,4]. The risk factors for all-cause mortality in AAV patients include the initial Birmingham Vasculitis Activity Score (BVAS), five-factor score (FFS), degree of inflammatory burden, and cumulative doses of immunosuppressive drugs and glucocorticoids, in addition to traditional risk factors in the general population, such as age, male sex, body mass index (BMI), smoking, and chronic metabolic diseases [5,6,7,8].

The total globulin fraction (TGF) is calculated by subtracting serum albumin levels from serum total protein levels and includes alpha-, beta-, and gamma-globulins [9]. As the majority of alpha-globulins are acute-phase reactants and gamma-globulins are immunoglobulins, TGF is expected to reflect the extent of various immune reactions as well as the degree of inflammatory burden [10]. Therefore, as previous studies have reported that the initial degree of inflammatory burden may affect all-cause mortality in AAV patients [5,7], it was assumed that initial TGF may forecast all-cause mortality during the disease course of AAV. However, there is no study regarding the predictive ability of TGF at AAV diagnosis for all-cause mortality in AAV patients to date. Hence, the present study examined whether TGF at AAV diagnosis could forecast all-cause mortality during the disease course in patients with MPA, GPA, and EGPA enrolled in a single-centre AAV cohort.

## 2. Materials and Methods

### 2.1. Patients

The present study included 283 patients with AAV (155 MPA, 72 GPA, and 56 EGPA) who were enrolled in the Severance Hospital ANCA-associated VasculitidEs (SHAVE) cohort. The SHAVE cohort is an observational cohort that includes patients who had been first diagnosed with AAV at the Division of Rheumatology, the Department of Internal Medicine, Yonsei University College of Medicine, Severance Hospital, from October 2000 to July 2020. All of the patients were first classified as having AAV at this tertiary university hospital. They had fulfilled both the 2007 European Medicine Agency algorithm for AAV and the 2022 revised Chapel Hill Consensus Conference nomenclature of AAV when enrolled in the cohort [1,2]. In addition, they were proved to be reclassified as AAV according to the 2012 American College of Rheumatology/European Alliance of Associations for Rheumatology classification criteria for AAV [11,12,13,14]. They had medical records in which clinical, laboratory, histological, and radiological data at the time of AAV diagnosis, particularly, regarding serum total protein and albumin levels as well as death, were clearly documented. Patients who had a follow-up duration ≥ three months after AAV diagnosis were included in the present study. However, patients with concomitant serious medical conditions such as malignancies and severe infectious diseases at AAV diagnosis were not registered in our AAV cohort [11]. Patients who had been classified as having AAV and further were concomitantly diagnosed with systemic autoimmune diseases after AAV diagnosis were excluded from the SHAVE cohort. In addition, patients who received immunosuppressive drugs for AAV treatment within 4 weeks before the confirmative diagnosis of AAV were also not enrolled in the SHAVE cohort. The present study was approved by the Institutional Review Board (IRB) of Severance Hospital (Seoul, Korea, IRB No. 4-2020-1071), and was conducted according to the Declaration of Helsinki. Given the retrospective design of the study and the use of anonymised patient data, the requirement for written informed consent was waived.

### 2.2. Clinical Data

The variables at AAV diagnosis and during the disease course are shown in Table 1. In the present study, myeloperoxidase (MPO)-ANCA and proteinase 3 (PR3)-ANCA were measured by an immunoassay, and perinuclear (P)-ANCA and cytoplasmic (C)-ANCA were identified by an indirect immunofluorescence assay to determine ANCA positivity [12,13,14]. BVAS and FFS were completed to assess AAV activity and prognosis indices [15,16]. Type 2 diabetes mellitus (T2DM), hypertension, and dyslipidaemia were considered comorbidities that concomitantly acted as risk factors for death [8,17,18,19]. TGF at AAV diagnosis was calculated by subtracting serum albumin levels from serum total protein levels at the time of AAV diagnosis [9,10]. For deceased patients, the follow-up duration based on all-cause mortality was defined as the period from AAV diagnosis to death. Meanwhile, for surviving patients, it was defined as the period from AAV diagnosis to the last visit. The number of patients who received glucocorticoids and immunosuppressive drugs during the disease course was counted.

### 2.3. Calculation of TGF

TGF (g/dL) = serum total protein (g/dL) − serum albumin (g/dL) [9].

### 2.4. Statistical Analyses

SPSS Statistics for Windows, version 26 (IBM Corp., Armonk, NY, USA) was used for all statistical analyses. Continuous and categorical variables are expressed as medians (25–75 percentiles) and numbers (percentages), respectively. The correlation coefficient (r) between the two variables was obtained using the Pearson correlation analysis. Significant differences between two continuous variables were compared using the Mann-Whitney U test. Significant differences among more than three continuous variables were investigated using the Kruskal-Wallis one-way analysis. The ROC curve was also used to obtain the appropriate cut-off value of TGF at AAV diagnosis for all-cause mortality. A comparison of the cumulative survivals rates between the two groups was performed using the Kaplan-Meier survival analysis with the log-rank test. The univariable and multivariable Cox hazards model analyses for all-cause mortality were performed to appropriately obtain the hazard ratios (HRs) during the considerable follow-up duration. A *p*-value < 0.05 was considered statistically significant.

## 3. Results

### 3.1. Characteristics of AAV Patients

Regarding the variables at AAV diagnosis, the median age of the 283 AAV patients was 60 years, and 35.7% were men. The median BMI was within the normal range and nine patients were ex-smokers. MPO-ANCA (or P-ANCA) and PR3-ANCA (or C-ANCA) were detected in 195 and 45 patients, respectively. The median BVAS, FFS, erythrocyte sedimentation rate (ESR), and C-reactive protein (CRP) levels were 12.0, 1.0, 56.0 mm/h, and 13.2 mg/L, respectively. The median TGF at AAV diagnosis was 2.9 (2.6–3.4). Of the 283 patients, 75, 114, and 58 patients had T2DM, hypertension, and dyslipidaemia, respectively. Regarding variables during the disease course, 39 patients (13.8%) died within a median follow-up duration of 46.9 months. Glucocorticoids were administered to 94.3% of patients, and the most frequently administered immunosuppressive drug was cyclophosphamide (55.5%), followed by azathioprine (52.7%) (Table 1).

### 3.2. Correlation Analysis

TGF at AAV diagnosis was not correlated with BVAS or FFS assessed at AAV diagnosis. However, TGF at AAV diagnosis was significantly correlated with ESR (r = 0.527, *p* < 0.001), and CRP levels (r = 0.358, *p* < 0.001) measured at AAV diagnosis (Figure S1). These findings suggest that TGF at AAV diagnosis might be associated with the cross-sectional acute-phase reactant levels, reflecting the degree of inflammatory burden rather than AAV activity or prognostic indices.

### 3.3. Comparison of TGF at AAV Diagnosis among the Groups

**Figure 1:**
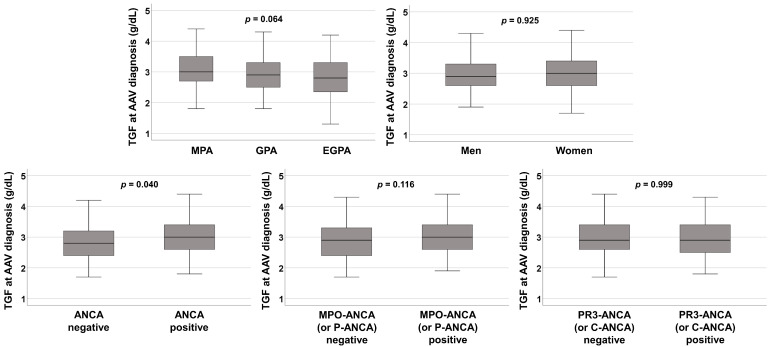
Comparative analysis of TGF at AAV diagnosis according to AAV subtype, sex, ANCA positivity, and ANCA type. TGF: the total globulin fraction; AAV: ANCA-associated vasculitis; ANCA: antineutrophil cytoplasmic antibody; MPA: microscopic polyangiitis; GPA: granulomatosis with polyangiitis; EGPA: eosinophilic granulomatosis with polyangiitis; MPO: myeloperoxidase; P: perinuclear; PR3: proteinase 3; C: cytoplasmic.

TGF at AAV diagnosis in patients with MPA or those with MPO-ANCA (or P-ANCA) positivity tended to be slightly elevated compared to that in patients with GPA and EGPA or those with MPO-ANCA (or P-ANCA) negativity (*p* = 0.064 and *p* = 0.116, respectively). However, the difference was not statistically significant. Also, TGF at AAV diagnosis did not differ between patients with PR3-ANCA (C-ANCA) and those without. There was no significant difference in TGF at AAV diagnosis based on the sex of the patients. Whereas, patients with ANCA positivity exhibited a significantly higher median TGF at AAV diagnosis than those with ANCA negativity (3.0 g/dL vs. 2.8 g/dL, *p* = 0.040) (Figure 1). These findings suggest that TGF at AAV diagnosis may be affected by the cross-sectional ANCA positivity and partially MPO-ANCA (or P-ANCA) positivity.

### 3.4. Optimal Cut-Off of TGF at AAV Diagnosis for All-Cause Mortality during Follow-Up

**Figure 2:**
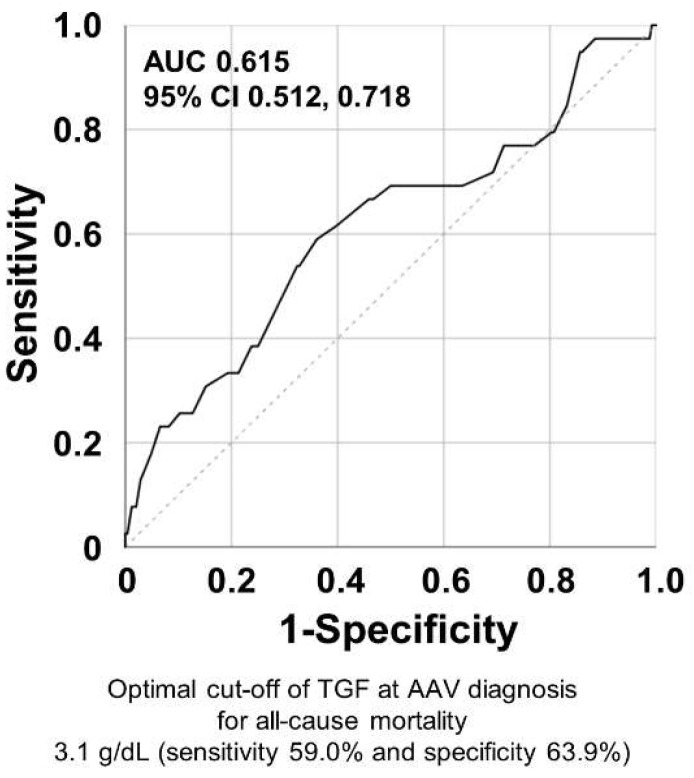
The ROC curve analysis for obtaining the cut-off of TGF for all-cause mortality. ROC: receiver operating characteristics; TGF: the total globulin fraction; AUC: area under the curve; AAV: ANCA-associated vasculitis; ANCA: antineutrophil cytoplasmic antibody.

When the ROC curve analysis of TGF at AAV diagnosis for all-cause mortality was performed, the area under the curve was 0.615 (95% confidence interval (CI) 0.512, 0.718). When the optimal cut-off of TGF at AAV diagnosis was set as 3.1 g/dL, the sensitivity, and specificity were 59.0% and 63.9%, respectively (Figure 2).

### 3.5. Comparison of Cumulative Patients’ Survival Rate according to the Cut-Off of TGF at AAV Diagnosis

**Figure 3:**
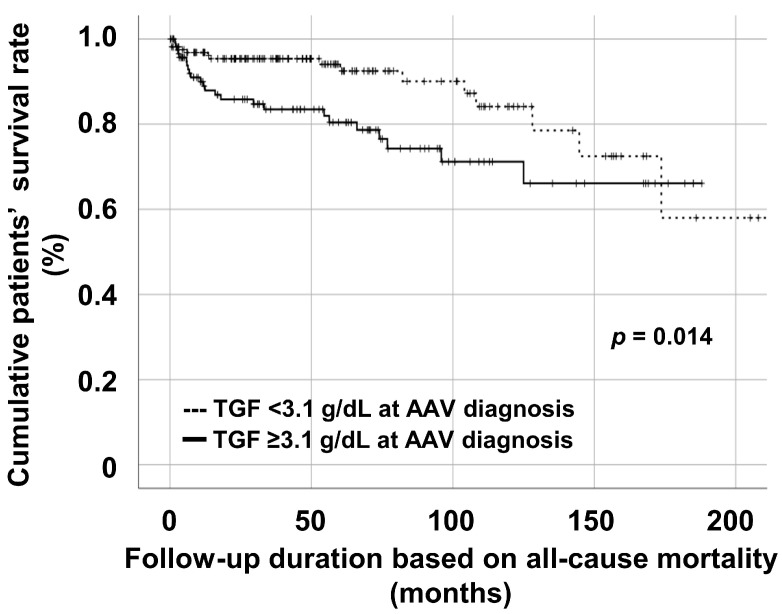
Comparative analysis of cumulative survival rates according to the cut-off of TGF for all-cause mortality. TGF: the total globulin fraction; AAV: ANCA-associated vasculitis; ANCA: antineutrophil cytoplasmic antibody.

Patients with TGF ≥ 3.1 g/dL at AAV diagnosis exhibited a significantly lower cumulative patients’ survival rate than those with TGF < 3.1 g/dL at AAV diagnosis (Figure 3).

### 3.6. Cox Hazards Model Analyses

In the univariable Cox analysis of the variables at AAV diagnosis, age (HR 1.063), male sex (HR 2.702), BMI (HR 1.122), BVAS (HR 1.082), FFS (HR 1.927), serum total protein (HR 0.590), serum albumin (HR 0.369), ESR (HR 1.010), CRP (HR 1.008), and TGF ≥ 3.1 g/dL (HR 2.197) were significantly associated with all-cause mortality during the disease course in AAV patients. Because TGF is calculated by subtracting serum albumin levels from serum total protein levels, serum total protein and albumin were excluded from the multivariable Cox analysis with TGF. In the multivariable Cox analysis, age (HR 1.037, 95% CI 1.002, 1.072), male sex (HR 3.136, 95% CI 1.495, 6.580), BMI (HR 1.114, 95% CI 1.001, 1.238), and TGF ≥ 3.1 g/dL (HR 2.611, 95% CI 1.223, 5.572) were significantly and independently associated with all-cause mortality during the disease course in AAV patients (Table 2).

## 4. Discussion

The present study examined the ability of TFG calculated at AAV diagnosis to forecast all-cause mortality during the disease course in AAV patients and results in the following findings. First, TGF at AAV diagnosis could reflect the cross-sectional degree of inflammatory burden represented by ESR and CRP rather than AAV activity [20]. Second, TGF at AAV diagnosis was affected by ANCA positivity at AAV diagnosis. Third, TGF at AAV diagnosis over its cut-off for all-cause mortality resulted in a lower patients’ survival rate during the disease course of AAV. Fourth, TGF at AAV diagnosis above its cut-off for all-cause mortality was independently associated with all-cause mortality during the disease course in AAV patients. Therefore, these findings suggest that TGF at AAV diagnosis may be useful in forecasting all-cause mortality during the disease course in AAV patients. This potential may enable us to cope with future mortality through closer monitoring [21].

**Figure 4:**
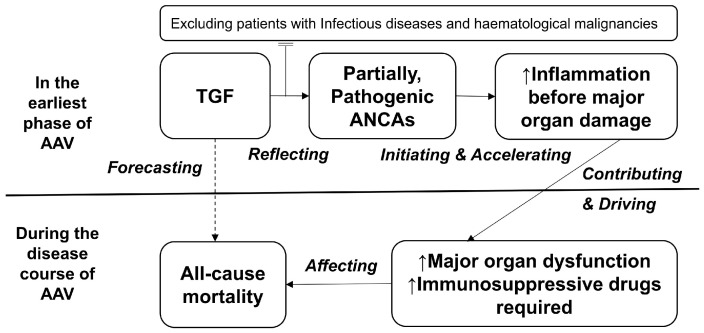
Hypothesis on the mechanism of how TGF at AAV diagnosis could forecast all-cause mortality during the disease course of AAV. TGF: the total globulin fraction; AAV: ANCA-associated vasculitis; ANCA: antineutrophil cytoplasmic antibody.

As patients with infectious diseases and immunoglobulin-producing haematological malignancies at AAV diagnosis were excluded from the present study according to the new classification criteria for AAV [11], a high level of TGF at AAV diagnosis might be associated with immunoglobulins that are produced due to dysregulated immune tolerance, such as autoantibodies [22]. In the present study, there was no significant difference in median TGF at AAV diagnosis between patients with and those without antinuclear antibodies (3.0 g/dL vs. 2.9 g/dL, *p* = 0.441). Conversely, patients with ANCA positivity exhibited a significantly higher median TGF at AAV diagnosis than those with ANCA negativity (Figure 1). Given that pathogenic ANCA participates in both phases, the neutrophil priming and activation phases in the pathogenesis of AAV [23,24], the following hypotheses can be put forward: (i) TGF at AAV diagnosis may reflect the amount of pathogenic ANCA at AAV diagnosis; (ii) the amount of ANCA at AAV diagnosis may affect the degree of inflammation in the earliest phase of AAV before major organ damage [25]; (iii) the degree of inflammation in the earliest phase of AAV before major organ damage may be associated with an increase in the rates of all-cause mortality owing to the increased requirement of immunosuppressive drugs and the frequent earlier major organ failure; and, (iv) therefore, TGF at AAV diagnosis may forecast all-cause mortality during the disease course in AAV patients (Figure 4).

In the multivariable Cox analysis of the present study, TGF ≥ 3.1 g/dL was a significant and independent risk factor for all-cause mortality in AAV patients, along with age, male sex, and BMI. The variables with a *p* value < 0.1 were accepted to be included in the multivariable Cox analysis, and dyslipidaemia was added to the Cox analysis. In this setting, age was no longer a significant risk factor for all-cause mortality. However, TGF ≥ 3.1 g/dL remained an independent risk factor for all-cause mortality in AAV patients, along with male sex, and BMI (Table S1). In addition, even when T2DM and hypertension, traditional risk factors for death in the general population [8], were included in the multivariable Cox analysis together with dyslipidaemia, TGF ≥ 3.1 g/dL did still remain an independent risk factor for all-cause mortality in AAV patients (Table S2). These findings suggest that the solid ability of TGF ≥ 3.1 g/dL at AAV diagnosis to forecast all-cause mortality during the disease course could be demonstrated in AAV patients.

Serum albumin levels are known as a serum inflammatory marker and their predictive potential for all-cause mortality in various disease has been proven [26,27]. In addition, we previously demonstrated that the ratio of serum albumin levels to TGF (albumin-globulin ratio [AGR]) at diagnosis is inversely associated with all-cause mortality in MPA patients [28]. We compared the abilities of these three variables, including TGF, albumin, and AGR at AAV diagnosis to forecast all-cause mortality in AAV patients using the ROC curve analysis. As serum albumin levels and AGR were inversely associated with all-cause mortality, the values calculated by subtracting them from 1 were used for the comparative analysis. The adjusted area under the curve values of 1-serum albumin levels and 1-AGR were significantly larger than that of TGF at diagnosis (0.615 [*p* = 0.021], 0.738 [*p* < 0.001], and 0.719 [*p* < 0.001], respectively) (Figure S2). Serum albumin levels are affected by diverse conditions such as substantial chronic liver diseases, serious malnutrition, and protein-losing enteropathy or nephropathies, in addition to the degree of inflammatory burden. Meanwhile, TGF is closely linked to immunoglobulin-related medical conditions such as autoimmune diseases compared to serum albumin levels [29,30]. Therefore, it can be concluded that, at least in AAV patients, TGF may have more reliable clinical implications in forecasting all-cause mortality than the remaining variables.

Since there were no multicollinearities between TGF and serum total protein or albumin in the multivariable linear regression analysis for death [31], it was feasible to include the two variables of serum total protein and albumin levels in the multivariable Cox analysis. We conducted the multivariable Cox analysis by including serum total protein and albumin levels again. As in Table 2, age, male sex, and BMI at AAV diagnosis were significantly and independently associated with all-cause mortality during the follow-up of AAV. In addition, FFS at AAV diagnosis was found to be a new independent predictor of all-cause mortality in AAV patients. Conversely, Conversely, although serum albumin at AAV diagnosis was strongly and independently associated with all-cause mortality during the disease course in AAV patients, TGF ≥ 3.1 g/dL at AAV diagnosis was not independently associated with all-cause mortality in AAV patients anymore (Table S3). However, given that serum albumin may be affected by various confounding factors mentioned above and the statistical strength in the correlation of TGF with serum total protein (r = 0.473, *p* < 0.001) and albumin (r = −0.256, *p* < 0.001) was too high to ignore, we thought it more reasonable to exclude those two variables from the multivariable Cox analysis in real clinical settings.

Although Figure 1 showed no significant differences among MPA, GPA, and EGPA patients, we wondered whether each AAV type might affect the frequency of death in AAV patients because the disease courses and prognoses may differ according to the AAV type. In the univariable Cox analysis, neither MPA (HR 1.656, 95% CI 0.860, 3.189, *p* = 0.131) nor GPA (HR 1.608, 95% CI 0.817, 3.164, *p* = 0.169) was not significantly associated with all-cause mortality during the disease course. Whereas, EGPA was inversely associated with all-cause mortality in AAV patients (HR 0.078, 95% CI 0.011, 0.574, *p* = 0.012). Therefore, we included EGPA in the multivariable Cox analysis and found that TGF ≥ 3.1 g/dL at AAV diagnosis still had the ability to forecast all-cause mortality during the disease course in AAV patients (HR 2.350, 95% CI 1.101, 5.014), along with age (HR 1.036), male sex (HR 2.815), and EGPA (HR 0.054) (Table S4).

The present study had several limitations. Most importantly, because the present study was conducted retrospectively, perfect real-time intervention or control was not possible. Moreover, because there are few cohorts of Korean patients with AAV, it was not possible to validate the results of the present study using patients of the same ethnicity and geographical background. These limitations may have decreased the reliability of the results of the present study. However, we believe that the present study has clinical implications as a pilot study, in that it is the first study to demonstrate the ability of TGF at AAV diagnosis to forecast all-cause mortality during the disease course in a considerable number of AAV patients who had no malignancies or severe infectious diseases at AAV diagnosis. We expect that a future prospective study with a larger number of patients will provide more reliable and clearer information on the role of TGF calculated at AAV diagnosis in forecasting all-cause mortality in AAV patients.

## 5. Conclusions

The present study is the first to demonstrate that TGF at AAV diagnosis can forecast all-cause mortality during the disease course in AAV patients. Based on our findings, we suggest that TGF should be calculated at AAV diagnosis and more attention should be paid to AAV patients when TGF calculated at AAV diagnosis is above the cut-off value.

## Figures and Tables

**Table 1:** Characteristics of AAV patients (N = 283).

Variables	Values
Variables at diagnosis	
Demographic data	
Age (years)	60.0 (49.0–69.0)
Male sex (N, (%))	101 (35.7)
BMI (kg/m^2^)	22.7 (20.3–24.6)
Ex-smoker (N, (%))	9 (3.2)
AAV subtype (N, (%))	
MPA	155 (54.8)
GPA	72 (25.4)
EGPA	56 (19.8)
ANCA type and positivity (N, (%))	
MPO-ANCA (or P-ANCA) positivity	195 (68.9)
PR3-ANCA (or C-ANCA) positivity	45 (15.9)
ANCA negativity	55 (19.4)
AAV-specific indices	
BVAS	12.0 (7.0–18.0)
FFS	1.0 (0–2.0)
Laboratory results	
White blood cell count (/mm^3^)	9280.0 (6420.0–13,010.0)
Haemoglobin (g/dL)	11.4 (9.5–13.2)
Platelet count (×1000/mm^3^)	294.0 (227.0–389.0)
Blood urea nitrogen (mg/dL)	17.7 (12.5–31.0)
Serum creatinine (mg/dL)	0.9 (0.7–1.8)
Serum total protein (g/dL)	6.6 (6.0–7.2)
Serum albumin (g/dL)	3.7 (3.1–4.2)
ESR (mm/h)	56.0 (21.5–95.0)
CRP (mg/L)	13.2 (1.6–64.9)
Total globulin fraction (g/dL)	2.9 (2.6–3.4)
Comorbidities (N, (%))	
T2DM	75 (26.5)
Hypertension	114 (40.3)
Dyslipidaemia	58 (20.5)
Variables during the disease course	
All-cause mortality (N, (%))	39 (13.8)
Follow-up duration based on all-cause mortality (months)	46.9 (16.8–79.0)
Medications (N, (%))	
Glucocorticoids	267 (94.3)
Cyclophosphamide	157 (55.5)
Rituximab	50 (17.7)
Mycophenolate mofetil	54 (19.1)
Azathioprine	149 (52.7)
Tacrolimus	24 (8.5)
Methotrexate	23 (8.1)

Values are expressed as a median (25–75 percentiles) or N (%). AAV: ANCA-associated vasculitis; ANCA: antineutrophil cytoplasmic antibody; BMI: body mass index; MPA: microscopic polyangiitis; GPA: granulomatosis with polyangiitis; EGPA: eosinophilic granulomatosis with polyangiitis; MPO: myeloperoxidase; P: perinuclear; PR3: proteinase 3; C: cytoplasmic; BVAS: Birmingham vasculitis activity score; FFS: five-factor score; T2DM: type 2 diabetes mellitus; ESR: erythrocyte sedimentation rate; CRP: C-reactive protein.

**Table 2:** Cox hazards model analyses of TGF at AAV diagnosis for all-cause mortality in AAV patients during the disease course.

Variables	Univariable			Multivariable		
	HR	95% CI	*p* Value	HR	95% CI	*p* Value
Age (years)	1.063	1.031, 1.095	<0.001	1.037	1.002, 1.072	0.036
Male sex (N, (%))	2.702	1.430, 5.103	0.002	3.136	1.495, 6.580	0.002
BMI (kg/m^2^)	1.122	1.029, 1.225	0.010	1.114	1.001, 1.238	0.047
Ex-smoker (N, (%))	1.619	0.389, 6.732	0.508			
MPO-ANCA (or P-ANCA) positivity	1.409	0.699, 2.843	0.338			
PR3-ANCA (or C-ANCA) positivity	0.693	0.271, 1.772	0.444			
BVAS	1.082	1.038, 1.128	<0.001	1.043	0.984, 1.107	0.156
FFS	1.927	1.423, 2.609	<0.001	1.359	0.951, 2.046	0.089
T2DM	1.083	0.547, 2.143	0.819			
Hypertension	1.129	0.600, 2.125	0.706			
Dyslipidaemia	1.808	0.915, 3.574	0.088			
White blood cell count (/mm^3^)	1.000	1.000, 1.000	0.047	1.000	1.000, 1.000	0.804
Haemoglobin (g/dL)	0.787	0.680, 0.911	0.001	0.932	0.740, 1.173	0.547
Platelet count (×1000/mm^3^)	1.000	0.998, 1.002	0.765			
Fasting glucose (mg/dL)	1.005	0.999, 1.010	0.112			
Blood urea nitrogen (mg/dL)	1.011	1.003, 1.019	0.005	1.000	0.985, 1.016	0.961
Serum creatinine (mg/dL)	1.139	1.014, 1.279	0.028	1.078	0.863, 1.347	0.508
ESR (mm/h)	1.010	1.002, 1.019	0.013	0.993	0.981, 1.006	0.295
CRP (mg/L)	1.008	1.004, 1.013	<0.001	1.004	0.997, 1.011	0.295
TGF ≥ 3.1 g/dL	2.197	1.152, 4.192	0.017	2.611	1.223, 5.572	0.013

Since TGF is calculated by subtracting serum albumin levels from serum total protein levels, the two variables were excluded from the multivariable Cox analysis with TGF. TGF: total globulin fraction; AAV: ANCA-associated vasculitis; ANCA: antineutrophil cytoplasmic antibody; BMI: body mass index; MPO: myeloperoxidase; P: perinuclear; PR3: proteinase 3; C: cytoplasmic; BVAS: Birmingham vasculitis activity score; FFS: five-factor score; T2DM: type 2diabetes mellitus; ESR: erythrocyte sedimentation rate; CRP: C-reactive protein.

## Data Availability

The data used to support the findings of this study are included within the article and the Supplementary Materials.

## Supplementary Materials

The following are available online at https://www.mdpi.com/article/10.3390/jcm12124170/s1, Figure S1: Correlation analyses of TGF at AAV diagnosis with BVAS, FFS, ESR, and CRP, Figure S2: Comparison of AUCs of TGF, serum albumin, and albumin-globulin ratio (AGR) for all-cause mortality in AAV patients, Table S1: Cox hazards model analyses of TGF at diagnosis for all-cause mortality in AAV patients during the disease course (including variables with *p* < 0.1 in the univariate analysis), Table S2: Cox hazards model analyses of TGF at diagnosis for all-cause mortality in AAV patients during the disease course (including traditional risk factors for all-cause mortality such as T2DM, hypertension and dyslipidaemia), Table S3: Cox hazards model analyses of TGF at AAV diagnosis for all-cause mortality in AAV patients during the disease course (including serum total protein and albumin), Table S4: Cox hazards model analyses of TGF at AAV diagnosis for all-cause mortality in AAV patients during the disease course (including EGPA).
