# Supplementary material for: Total Globulin Fraction at Diagnosis Could Forecast All-Cause Mortality during the Disease Course in Patients with Antineutrophil Cytoplasmic Antibody-Associated Vasculitis
Source: J Clin Med. 2023 Jun 20;12(12):4170. doi: 10.3390/jcm12124170 (PMC10298886; doi:10.3390/jcm12124170)
Supplement: Supplementary file 1 [file jcm-12-04170-s001.zip › Table S1.pdf]

Table S1. Cox hazards model analyses of TGF at diagnosis for all-cause mortality in AAV patients during the disease course (including variables with *p* < 0.1 in the univariate analysis)

Variables	Univariable			Multivariable		
	**HR**	**95% CI**	***p* value**	**HR**	**95% CI**	***p* value**
Age (years)	1.063	1.031, 1.095	<0.001	1.033	0.998, 1.068	0.065
Male sex (N, (%))	2.702	1.430, 5.103	0.002	3.288	1.559, 6.937	0.002
BMI (kg/m^2)	1.122	1.029, 1.225	0.010	1.118	1.004, 1.245	0.043
Ex-smoker (N, (%))	1.619	0.389, 6.732	0.508			
MPO-ANCA (or P-ANCA) positivity	1.409	0.699, 2.843	0.338			
PR3-ANCA (or C-ANCA) positivity	0.693	0.271, 1.772	0.444			
BVAS	1.082	1.038, 1.128	<0.001	1.051	0.992, 1.114	0.092
FFS	1.927	1.423, 2.609	<0.001	1.413	0.967, 2.066	0.074
T2DM	1.083	0.547, 2.143	0.819			
Hypertension	1.129	0.600, 2.125	0.706			
Dyslipidaemia	1.808	0.915, 3.574	0.088	1.765	0.807, 3.858	0.155
White blood cell count ($/mm^3$)	1.000	1.000, 1.000	0.047	1.000	1.000, 1.000	0.602
Haemoglobin (g/dL)	0.787	0.680, 0.911	0.001	0.934	0.743, 1.175	0.561
Platelet count ($\times$ $1000/mm^3$)	1.000	0.998, 1.002	0.765			
Fasting glucose (mg/dL)	1.005	0.999, 1.010	0.112			
Blood urea nitrogen (mg/dL)	1.011	1.003, 1.019	0.005	1.000	0.984, 1.015	0.976
Serum creatinine (mg/dL)	1.139	1.014, 1.279	0.028	1.090	0.873, 1.362	0.446
ESR (mm/hr)	1.010	1.002, 1.019	0.013	0.995	0.982, 1.007	0.389
CRP (mg/L)	1.008	1.004, 1.013	<0.001	1.003	0.996, 1.010	0.448
TGF ≥3.1 g/dL	2.197	1.152, 4.192	0.017	2.546	1.196, 5.418	0.015

TGF: total globulin fraction; AAV: ANCA-associated vasculitis; ANCA: antineutrophil cytoplasmic antibody; BMI: body mass index; MPO: myeloperoxidase; P: perinuclear; PR3: proteinase 3; C: cytoplasmic; BVAS: Birmingham vasculitis activity score; FFS: five-factor score; T2DM: type 2diabetes mellitus; ESR: erythrocyte sedimentation rate; CRP: C-reactive protein.
