# Supplementary material for: Total Globulin Fraction at Diagnosis Could Forecast All-Cause Mortality during the Disease Course in Patients with Antineutrophil Cytoplasmic Antibody-Associated Vasculitis
Source: J Clin Med. 2023 Jun 20;12(12):4170. doi: 10.3390/jcm12124170 (PMC10298886; doi:10.3390/jcm12124170)
Supplement: Supplementary file 1 [file jcm-12-04170-s001.zip › Table S2.pdf]

**Table S2. Cox hazards model analyses of TGF at diagnosis for all-cause mortality in AAV patients during the disease course (including traditional risk factors for all-cause mortality such as T2DM, hypertension and dyslipidaemia)**

| Variables | Univariable | | | Multivariable | | |
|---|---|---|---|---|---|---|
| | HR | 95% CI | *p* value | HR | 95% CI | *p* value |
| Age (years) | 1.063 | 1.031, 1.095 | <0.001 | 1.032 | 0.998, 1.068 | 0.064 |
| Male sex (N, (%)) | 2.702 | 1.430, 5.103 | 0.002 | 3.142 | 1.453, 6.793 | 0.004 |
| BMI ($kg/m^2$) | 1.122 | 1.029, 1.225 | 0.010 | 1.123 | 1.007, 1.251 | 0.037 |
| Ex-smoker (N, (%)) | 1.619 | 0.389, 6.732 | 0.508 | | | |
| MPO-ANCA (or P-ANCA) positivity | 1.409 | 0.699, 2.843 | 0.338 | | | |
| PR3-ANCA (or C-ANCA) positivity | 0.693 | 0.271, 1.772 | 0.444 | | | |
| BVAS | 1.082 | 1.038, 1.128 | <0.001 | 1.050 | 0.990, 1.113 | 0.102 |
| FFS | 1.927 | 1.423, 2.609 | <0.001 | 1.422 | 0.972, 2.079 | 0.070 |
| T2DM | 1.083 | 0.547, 2.143 | 0.819 | 0.707 | 0.319, 1.567 | 0.393 |
| Hypertension | 1.129 | 0.600, 2.125 | 0.706 | 0.902 | 0.428, 1.903 | 0.787 |
| Dyslipidaemia | 1.808 | 0.915, 3.574 | 0.088 | 2.068 | 0.875, 4.885 | 0.098 |
| White blood cell count ($/mm^3$) | 1.000 | 1.000, 1.000 | 0.047 | 1.000 | 1.000, 1.000 | 0.628 |
| Haemoglobin (g/dL) | 0.787 | 0.680, 0.911 | 0.001 | 0.944 | 0.752, 1.186 | 0.623 |
| Platelet count ($\times 1000/mm^3$) | 1.000 | 0.998, 1.002 | 0.765 | | | |
| Fasting glucose (mg/dL) | 1.005 | 0.999, 1.010 | 0.112 | | | |
| Blood urea nitrogen (mg/dL) | 1.011 | 1.003, 1.019 | 0.005 | 1.000 | 0.985, 1.016 | 0.984 |
| Serum creatinine (mg/dL) | 1.139 | 1.014, 1.279 | 0.028 | 1.085 | 0.868, 1.355 | 0.473 |
| ESR (mm/hr) | 1.010 | 1.002, 1.019 | 0.013 | 0.994 | 0.982, 1.007 | 0.375 |
| CRP (mg/L) | 1.008 | 1.004, 1.013 | <0.001 | 1.003 | 0.996, 1.011 | 0.422 |
| TGF ≥3.1 g/dL | 2.197 | 1.152, 4.192 | 0.017 | 2.732 | 1.263, 5.911 | 0.011 |

TGF: total globulin fraction; AAV: ANCA-associated vasculitis; ANCA: antineutrophil cytoplasmic antibody; BMI: body mass index; MPO: myeloperoxidase; P: perinuclear; PR3: proteinase 3; C: cytoplasmic; BVAS: Birmingham vasculitis activity score; FFS: five-factor score; T2DM: type 2diabetes mellitus; ESR: erythrocyte sedimentation rate; CRP: C-reactive protein.
