# Supplementary material for: Total Globulin Fraction at Diagnosis Could Forecast All-Cause Mortality during the Disease Course in Patients with Antineutrophil Cytoplasmic Antibody-Associated Vasculitis
Source: J Clin Med. 2023 Jun 20;12(12):4170. doi: 10.3390/jcm12124170 (PMC10298886; doi:10.3390/jcm12124170)
Supplement: Supplementary file 1 [file jcm-12-04170-s001.zip › Table S4.pdf]

Table S4. Cox hazards model analyses of TGF at AAV diagnosis for all-cause mortality in AAV patients during the disease course (including EGPA)

Variables	Univariable			Multivariable		
	HR	95% CI	*p* value	HR	95% CI	*p* value
Age (years)	1.063	1.031, 1.095	<0.001	1.036	1.000, 1.074	0.048
Male sex (N, (%))	2.702	1.430, 5.103	0.002	2.815	1.347, 5.880	0.006
BMI (kg/m^2)	1.122	1.029, 1.225	0.010	1.084	0.971, 1.209	0.151
Ex-smoker (N, (%))	1.619	0.389, 6.732	0.508			
EGPA	0.078	0.011, 0.574	0.012	0.054	0.006, 0.468	0.008
MPO-ANCA (or P-ANCA) positivity	1.409	0.699, 2.843	0.338			
PR3-ANCA (or C-ANCA) positivity	0.693	0.271, 1.772	0.444			
BVAS	1.082	1.038, 1.128	<0.001	1.060	0.999, 1.124	0.052
FFS	1.927	1.423, 2.609	<0.001	1.463	0.988, 2.167	0.058
T2DM	1.083	0.547, 2.143	0.819			
Hypertension	1.129	0.600, 2.125	0.706			
Dyslipidaemia	1.808	0.915, 3.574	0.088			
White blood cell count (/mm^3)	1.000	1.000, 1.000	0.047	1.000	1.000, 1.000	0.161
Haemoglobin (g/dL)	0.787	0.680, 0.911	0.001	1.022	0.813, 1.285	0.850
Platelet count (× 1000/mm^3)	1.000	0.998, 1.002	0.765			
Fasting glucose (mg/dL)	1.005	0.999, 1.010	0.112			
Blood urea nitrogen (mg/dL)	1.011	1.003, 1.019	0.005	0.997	0.982, 1.013	0.734
Serum creatinine (mg/dL)	1.139	1.014, 1.279	0.028	1.102	0.876, 1.386	0.406
ESR (mm/hr)	1.010	1.002, 1.019	0.013	0.993	0.980, 1.006	0.276
CRP (mg/L)	1.008	1.004, 1.013	<0.001	1.004	0.996, 1.012	0.317
TGF ≥3.1 g/dL	2.197	1.152, 4.192	0.017	2.350	1.101, 5.014	0.027

Because TGF is calculated by subtracting serum albumin levels from serum total protein levels, the two variables were excluded from the multivariable Cox analysis with TGF.

TGF: total globulin fraction; AAV: ANCA-associated vasculitis; ANCA: antineutrophil cytoplasmic antibody; BMI: body mass index; MPO: myeloperoxidase; P: perinuclear; PR3: proteinase 3; C: cytoplasmic; BVAS: Birmingham vasculitis activity score; FFS: five-factor score; T2DM: type 2diabetes mellitus; ESR: erythrocyte sedimentation rate; CRP: C-reactive protein.
