# Supplementary figures and images for: Total Globulin Fraction at Diagnosis Could Forecast All-Cause Mortality during the Disease Course in Patients with Antineutrophil Cytoplasmic Antibody-Associated Vasculitis
Source: J Clin Med. 2023 Jun 20;12(12):4170. doi: 10.3390/jcm12124170 (PMC10298886; doi:10.3390/jcm12124170)

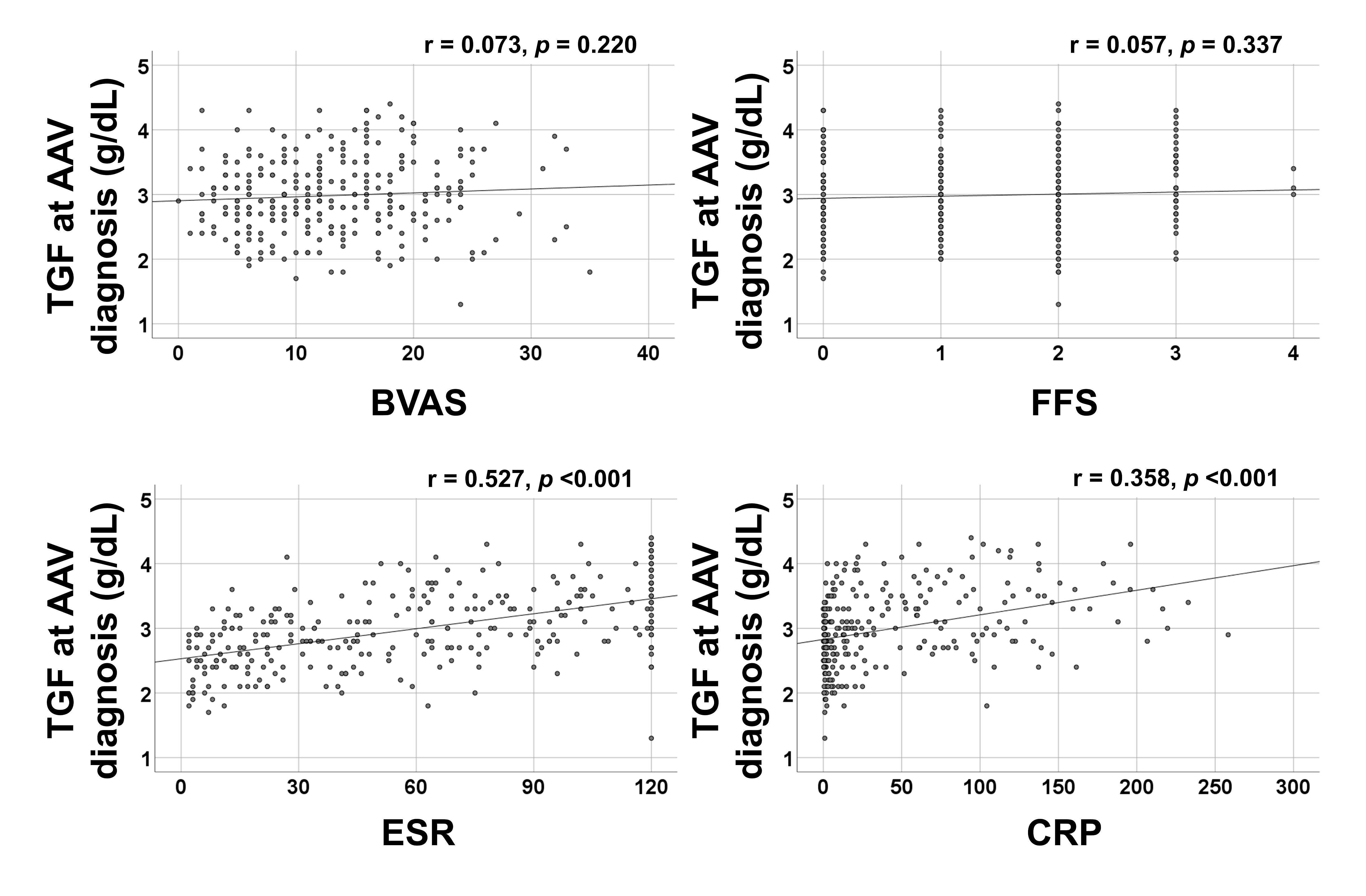

Supplement: Supplementary file 1 [file jcm-12-04170-s001.zip › Figure S1.tif]

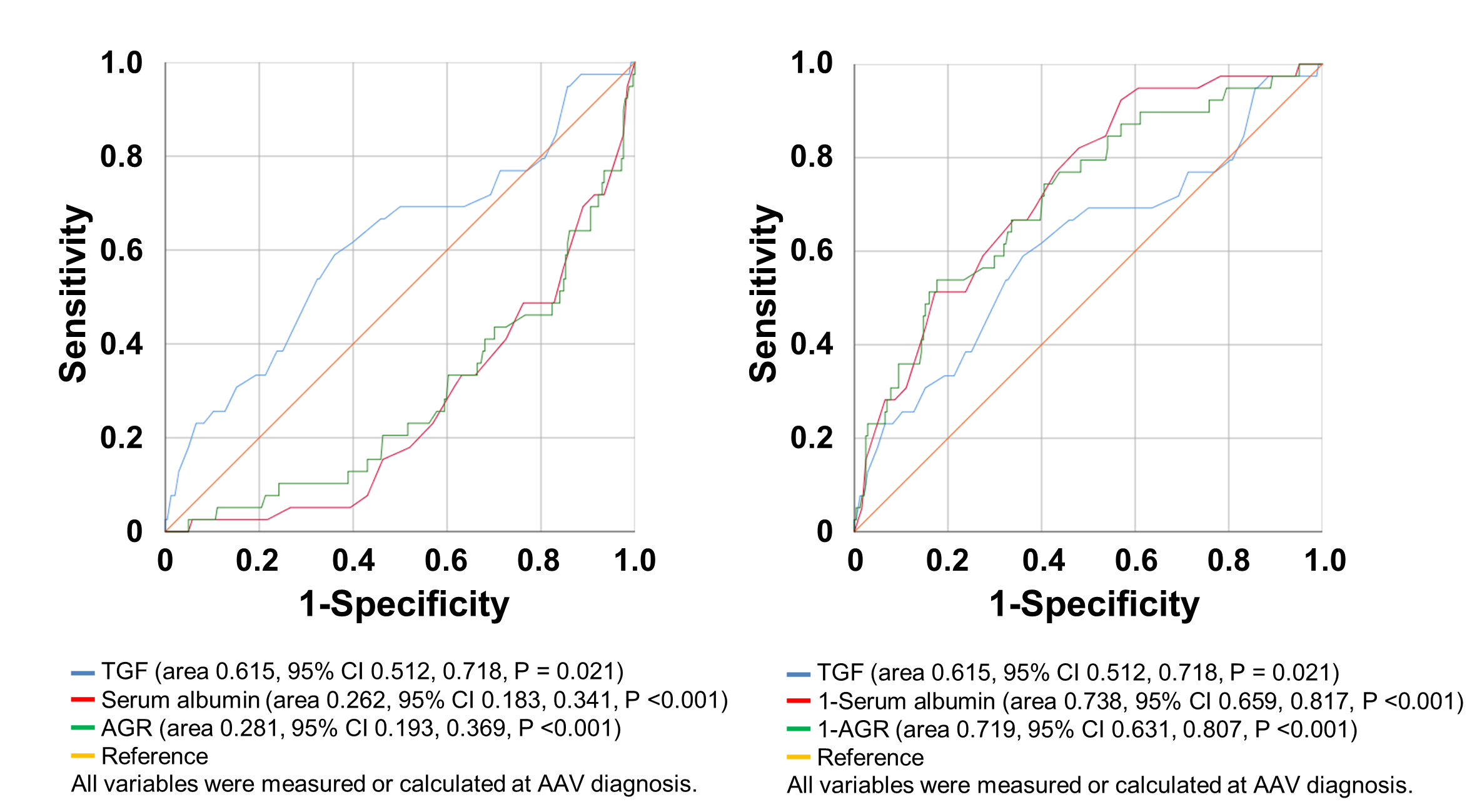

Supplement: Supplementary file 1 [file jcm-12-04170-s001.zip › Figure S2.tif]
